# Miscellaneous Cases

**Published:** 1828-09

**Authors:** 


### MISCELLANEOUS CASES.

*A Case of Rupture of the Bladder, with Separation of the Symphysis Pubis. Death sixty hours after the occurrence of the accident.*

David Hill, æt. thirty-five, horse-breaker, a stout, powerful man, was brought to the Worcester Infirmary, on the 5th of June, in the evening, having met with an accident, from a colt rearing up and falling upon him, about three hours before. His face was pallid and distressed; pulse very feeble. He complained of great pain about the left hip, particularly on motion. He had not passed any urine since the accident, or for three hours before.

The person who brought him said that, at the time of the accident, he was intoxicated.

A gum catheter was passed, and left in the bladder: about a pint of bloody urine was drawn off.—He was ordered half an ounce of brandy every two hours.

June 6th.—The pulse had got up. There was very extensive ecchymosis over the pubes, and both groins; great tenderness and tension over the abdomen; no stool; vomiting very frequent.

Appl$^{r}$ Hirud. xxx. abdom.—℞. Hyd. Submur. gr. x.; Pulv. Jalap. gr. x. fiat bol. j. stat. sum.—Mist. Cath. quartis horis.—Adhib. Enema vespere.

7th.—Vomiting continues of dark-coloured matter; pulse hardly perceptible; intellect clear; no stool; urine flows through the catheter. Tension and tenderness of the abdomen increased.

8th.—Died this morning.

*Sectio cadaveris.*—Integuments, and the whole of the abdomen below the umbilicus, much contused. The symphisis pubis was separated throughout its whole extent, so as to allow the thumb to be introduced between the bones. The bladder was ruptured transversely through the fundus and peritoneum covering it, to the extent of about four inches. A small quantity of urine, mixed with pus, was discovered in the pelvis. The intestines shewed patches of inflammation here and there; and about three inches of the intestinum ilium was quite black, from ecchymosis between its coats.

---

*Case of Wounded Liver from a Gun-shot Wound. Death in seventeen hours.*

James Banks, a stout young man, about sixteen years of age, was brought to the Worcester Infirmary, on the 5th of November, about six o'clock in the evening. He had been firing off an old cannon or pistol barrel, charged with a quantity of powder and gravel, which burst, and struck him down. There was a small wound, just large enough to admit the little finger, over the cartilages of the false ribs on the right side, about three inches from the ensiform cartilage. His face was extremely pallid; pulse small and fluttering; breathing very laborious. Blood flowed in considerable quantity from the wound, dark in colour, and thin, as if diluted with other liquid. He gradually sunk, and died about eleven o'clock the next morning.

*Sectio cadaveris.*—The cartilages of the false ribs, under the wound, were much shattered; a wound, about an inch in length, was discovered in the liver, close to the ligamentum suspensorium, passing through its substance, and coming out on the left side of the gallbladder; the first portion of the duodenum, close to the pylorus, was laid open to some extent. The pancreas was also wounded; and a piece of iron, about two inches in length, and an inch in breadth, much bent, with jagged edges, was found lodged in its substance. The right hepatic artery was wounded, also the hepatic vein: this latter vessel, from the appearance of the blood, chiefly furnished the hemorrhage. A good deal of coagulated blood, mixed with the contents of the duodenum, was found in the cavity of the abdomen.

---

*Misplaced Colon, with Enteritis.*

October, 1823.—Ann B. had been, two years before, a patient in the Infirmary, under my care, with a fixed tumor in the right iliac region, painful and tender. The bowels were then costive, the abdomen tense, the tongue foul, the pulse rapid and weak, and the emaciation great. She was ordered a seton over the situation of the tumor, occasional doses of oleum ricini, and low diet. Under this treatment the tumor nearly disappeared, the other symptoms yielded; and she gradually regained flesh and strength.

After a violent fit of anger, in which she used great muscular exertion, she was suddenly seized with fixed and violent pain in the bowels, of true inflammatory character. The bowels were at first loose, but afterwards obstinately bound, with vomiting of bilious matter. The seat of the old disease was the most tender part of the abdomen.

She was bled generally and locally, largely and repeatedly. In seventy-two hours from the seizure, the abdomen became tympanitic, the pulse too rapid to be counted, and she soon afterwards expired.

*Sectio cadaveris*, thirty-six hours after death.—The omentum, at its lower edge, was highly inflamed, and adherent, in the right iliac region, to the peritoneal lining of the abdominal muscles, and to the subjacent intestines. A portion of it was sphacelated. The portion of intestine with which it was chiefly connected proved to be the centre of the transverse arch of the colon, misplaced, and having its peritoneal coat thickened nearly to a quarter of an inch, over a space as large as three half-crowns: for the colon, instead of passing across the epigastric region, descended from the right hypochondriac region to the right iliac, and then ascended again to the left hypochondriac region, whence it pursued its usual course.

The diseased portion of the colon had part of its peritoneal coat in a state of sphacelus; but the villous coat was healthy at this part, and throughout the whole tract of the intestines. The diseased part of the colon was adherent to the cæcum below, but the cæcum was free from disease. There was a soft albuminous deposit on the folds of the intestines, and on the under surface of the omentum; there was also about a pint of serum in the cavity of the peritoneum.

*Remarks.*—The diseased portion of the intestine, which evidently constituted the original tumor, was, from its situation, considered, by all who saw her when she was in the Infirmary, to be the cæcum. The fact of the displacement of the transverse arch could not be ascertained during the life-time of the patient. This case shews how rapid and severe is acute inflammatory action, when it takes place in a morbidly altered structure, in which perhaps chronic inflammation is going on. It shews, too, that those violent passions of the mind, which augment generally the force and rapidity of arterial action, are powerful exciting causes of local inflammation.

---

### *Hydrocephalus Acutus.*

In a child, aged six years, who died with all the symptoms of this disease, I found more than two ounces of fluid beneath the tunica arachnoidea, but not any in the ventricles of the brain. The brain was highly vascular, and very hard.

In this case, in the early part of the first stage, vomiting was produced by sudden noises, as a clock striking. This I witnessed more than once.

---

### *Case of Tumor in the Uterus.*

In August, 1813, Susan Turberville, fifty-three years of age, was made an in-patient of Worcester Infirmary, with an ulcer in the left leg, which had existed for five months. In addition to this there had been, for some years, great enlargement of the abdomen. It was as prominent as in the sixth month of pregnancy. The enlargement was general over the lower part of the abdomen, not greater on one side than the other, extending from the pubes nearly up to the umbilicus. She was generally in pain about these parts, and in the groins; there was likewise tenderness on pressure. The appetite was good, but she was troubled with wind on the stomach. The bowels were always costive. She had frequent micturition, and always pain in evacuating the bladder. She was quite incapable of any active employment, but able to gain her livelihood by knitting, though she could not sit long in one place, being easier for gentle exercise. The breath was always short, but there was no cough. She had never borne children. The catamenia flowed till the usual period. The leg was cured by the latter end of December, and she went away from the infirmary, much in the same state as when admitted with respect to her visceral disease.

The leg continued well for about a twelvemonth, but the disease in the abdomen kept gradually and slowly increasing.

On the 16th of September, 1815, she was again admitted into the infirmary, on account of a small ulcer on the inner ankle of the right leg. The visceral disease had evidently gained much ground. The pulse was now always hard, and quicker than natural; the pain at the lower part of the abdomen was much greater; there was more tenderness on pressure, and the belly was increased in size; and the breath was much shorter. There was also a constant, profuse, thick, white discharge per vaginam. The bowels were more costive, and micturition was more painful. The stomach was much oppressed with flatus, but the appetite still continued good.

On the 16th of October, she had the following pills directed for her:

℞. Ferri Sulphatis, gr. iij.; Gummi Olibani, gr. x.; Cons. q. s. fiat pilulæ ij. bis die sumendæ.

The intention with which these pills were given was to check the discharge, and give tone to the stomach. At the end of a week from taking the pills, the discharge was much lessened, but in other respects she felt much as before. By the 20th of October, the discharge had almost ceased; but she was, in other respects, the same as when admitted into the infirmary.

On the 28th, she complained of being worse, having rather more pain over the abdomen, and great pain in the head. At night she was incoherent, wandering from one subject to another. The bowels had not been moved for two days. Some calomel and antimonial powder were taken at night, and a blister was applied to the nape of the neck. The night, was restless; but the bowels were moved very freely on the morning of the 29th.

On the 30th, she began to complain of great increase of pain in the abdomen, with tension and much tenderness on pressure. No vomiting; pulse hard, small and wiry. It was found, on inquiry, she had an umbilical hernia; but, on examination, it did not appear strangulated. Ten leeches were applied to the abdomen, by which she appeared slightly relieved, but shortly after the tension, pain, and tenderness returned. The countenance now became ghastly, and the eyes sunk; she had constant tenesmus. A large blister was applied to the abdomen, and she had an opiate enema. At about ten o'clock that night she vomited for the first time, which recurred several times in the night. There was no relief from the blister, and all the direful train of symptoms which usually characterise the last stage of peritoneal inflammation now shewed themselves.

She died at three o'clock of the morning of the 31st.

*Examination of the body*, twenty-four hours after death.—On opening the abdomen, purulent matter was observed covering all the intestines. The uterus appeared to occupy the same space in the abdomen that it does in the seventh month of pregnancy: it was firmly and closely connected anteriorly with the peritoneal lining of the abdominal muscles. At the posterior part, it was connected with the peritoneal covering of the bowels, and, as in the healthy state, with the sides of the pelvis, by the ligamenta lata, as also to the pudendum, by the ligamenta rotunda. On cutting it from its connexions with the above-mentioned parts, and elevating it, a process was found descending from the lower and posterior part of this large uterus into the cavity of the pelvis, passing between the rectum and the os sacrum, and separating the former from the latter. The vagina and the urethra were naturally situated. The whole surface of the tumor was very much inflamed, and covered with pus. The peritoneal lining of the abdominal muscles was in the same state. The colon was larger than natural, but not distended with fecal matter. The cæcum was large. The rectum not larger than natural. The small intestines of their usual size.

The peritoneal covering of the stomach was much inflamed ; its upper surface adhering, by recent lymph, to the under surface of the left lobe of the liver. The liver was twice its natural size; its peritoneal coat inflamed, recent lymph being deposited on it. In addition to this, it was firmly and closely united, by old adhesion, to the diaphragm. The spleen enlarged, and very soft in its structure; its peritoneal coat had become cartilaginous in some places. Pancreas healthy. Kidneys healthy. Urinary bladder full of small calculi. Thoracic viscera healthy.

There was nothing but omentum contained in the hernial sac at the umbilicus, and it was not strangulated. On macerating the diseased parts, it was found that a tumor had grown within the uterus, much resembling the muscular structure of that organ. The uterus could with ease be separated from the tumor, being connected with it by common cellular substance.

The uterus and tumor together weighed 14℔. 8½oz.

*Observations.*—It is remarkable in this case that two very important organs, the uterus and the liver, were very considerably diseased, and yet this woman, until within a very short period of her death, appeared to suffer but little in her general health : there was, indeed, no other symptom of constitutional disturbance, but a difficulty in passing the urine and constipation.

Dr. Baillie seems to regard growths of this description in the uterus as tubercles : he says, " A mass of the same kind is sometimes found in the cavity of the uterus, and often grows to a very large size : I have seen it a good deal larger than a child's head at birth. This mass, when cut into, exhibits precisely the same appearances as those which we have so lately described. It is remarkable that such masses within the cavity of the uterus commonly do not adhere in any part closely to it, but are connected with it loosely, by the intervention of cellular membrane and small blood-vessels, so that they can be very easily peeled off without injuring the structure of the uterus."—" These tubercles," he says in another place, "have a structure much resembling that of the uterus itself."

The size of the tumor, in the case above related, is greater than any one alluded to in Baillie's works, as he mentions one the size of a child's head at birth as the largest he had seen, which falls far short of the one here detailed.*

* The Midland Medical and Surgical Reporter, No. 1.

